# Study protocol of the multicentre, randomised, triple-blind, placebo-controlled MERCURI-2 trial: promoting effective renoprotection in cardiac surgery patients by inhibition of sodium glucose cotransporter (SGLT)-2

**DOI:** 10.1136/bmjopen-2024-095504

**Published:** 2025-05-16

**Authors:** Maartina Oosterom-Eijmael, Nelson P Monteiro de Oliveira, Ed D Niesten, Martijn Tolsma, Ferdinand TF Snellen, Bas M Gerritse, Thierry V Scohy, T Rettig, M B Godfried, Magiel F Voogd, Jeroen Wink, Lisa MM van der Werff, Susanne Eberl, Benedikt Preckel, Jeroen Hermanides, Daniel H van Raalte, Abraham H Hulst, The MERCURI-2 study group, Marcel G Dijkgraaf

**Affiliations:** 1 Department of Anesthesiology, Amsterdam University Medical Center, Amsterdam, The Netherlands; 2 Department of Endocrinology, Amsterdam University Medical Center, Amsterdam, The Netherlands; 3 Amsterdam Cardiovascular Sciences Research Institute, Amsterdam, The Netherlands; 4 Department of Anesthesiology, Medisch Spectrum Twente, Enschede, The Netherlands; 5 Department of Anesthesiology and Intensive Care, Isala Clinics, Zwolle, The Netherlands; 6 Anaesthesiology, Amphia Ziekenhuis, Breda, North Brabant, The Netherlands; 7 Anesthesiology, Intensive Care and Pain Medicine, Amphia Hospital site Molengracht, Breda, Noord-Brabant, The Netherlands; 8 Department of Anesthesiology, Onze Lieve Vrouwe Gasthuis, Amsterdam, Noord-Holland, The Netherlands; 9 Department of Anesthesiology, Medisch Centrum Leeuwarden, Leeuwarden, The Netherlands; 10 Department of Anesthesiology, Leiden University Medical Center, Leiden, The Netherlands

**Keywords:** Cardiac surgery, Randomized Controlled Trial, acute kidney injury

## Abstract

**Introduction:**

Acute kidney injury (AKI) is a major complication after cardiac surgery and is associated with postoperative morbidity and mortality. Currently, no effective therapy exists to reduce the incidence of postoperative AKI. Sodium-glucose cotransporter-2 (SGLT2) inhibitors are effective in reducing AKI in outpatient settings for patients with chronic kidney disease. We hypothesised that perioperative SGLT2 inhibition will also reduce AKI incidence after cardiac surgery according to Kidney Disease: Improving Global Outcomes (KDIGO) criteria.

**Methods and analysis:**

We designed a multicentre, randomised, placebo-controlled, triple-blinded, superiority trial. A total of 784 patients, aged above 18 years, undergoing cardiac surgery will be included with stratification for sex and type 2 diabetes in a 1:1 ratio. Patients will receive either dapagliflozin 10 mg or placebo from the day before until 2 days after surgery. Serum creatinine will be measured preoperatively and daily for the first 7 days after the operation, and urine output will be measured until the urinary catheter is removed. The primary outcome is the incidence of postoperative AKI according to the KDIGO criteria.

**Ethics and dissemination:**

The medical ethics committee of the Amsterdam University Medical Centre (UMC) and the Dutch competent authority approved the study protocol (currently, version 9, 19 January 2024). This is an investigator-initiated study. The Amsterdam UMC, as sponsor, retains ownership of all data and publication rights. After completion of the trial, results will be disseminated to participants, patient societies and physicians via a network meeting and digital newsletter. Results will be submitted for publication in a peer-reviewed international medical journal and presented on (inter)national congresses.

**Trial registration number:**

Clinicaltrials.gov identifier: NCT05590143.

**STRENGTHS AND LIMITATIONS OF THIS STUDY**

- A randomised controlled trial using sodium-glucose cotransporter-2 inhibitors to prevent acute kidney injury (AKI) after cardiac surgery.
- Multicentre, randomised, triple-blind and placebo-controlled.
- An easily clinically implementable intervention starting only 1 day prior to the operation.
- Repurposing of a widely-used and safe kidney protective drug.
- As postoperative AKI is the primary outcome in this study, further research is required to investigate the longer-term impact.

## Introduction

 Annually, more than 2 million cardiac surgeries are performed worldwide, and the incidence of cardiac surgery-associated acute kidney injury (CSA-AKI) is reported to be up to 70%.^1^ Currently, no evidence-based preventive options are available.^2^ The reported incidence varies considerably between countries and centres, which may depend on standards of surgery as well as perioperative care, selected patient population, type of surgery and, perhaps most importantly, disparities in AKI definition and monitoring. The majority (71%) of diagnosed cases of CSA-AKI are classified as stage 1, according to Kidney Disease: Improving Global Outcomes (KDIGO) criteria, which include an increase in serum creatinine of ≥0.3 mg/dL within 48 hours, ≥ 1.5 times baseline within 7 days or urine output <0.5 mL/kg/h for 6–12 hours and resolve without treatment or medical intervention.^3^ However, 12% of cases fall into stage 3, potentially necessitating renal replacement therapy for the management of AKI.^3^ CSA-AKI is independently associated with higher postoperative mortality compared with patients without AKI: 3 times higher mortality for patients with stage 1 AKI and up to 37 times higher for patients requiring dialysis (stage 3 AKI). Patients with AKI also have enhanced length of stay in the intensive care unit (ICU) and hospital and marked increments in cost of care, ranging from 1.21 times higher costs for stage 1 AKI to 2.74 for stage 3 AKI requiring dialysis.^2 4^ In addition, patients with only stage 1 AKI have a higher probability of developing chronic kidney disease (CKD) in the years following surgery, as compared with individuals without postoperative AKI.^5^ The risk of death associated with AKI remains increased for up to 10 years after cardiac surgery, even for those with complete recovery of kidney function.^4 6^ It is evident from these data that CSA-AKI represents a major unmet medical need posing a burden on patients and society.

**Figure 1:**
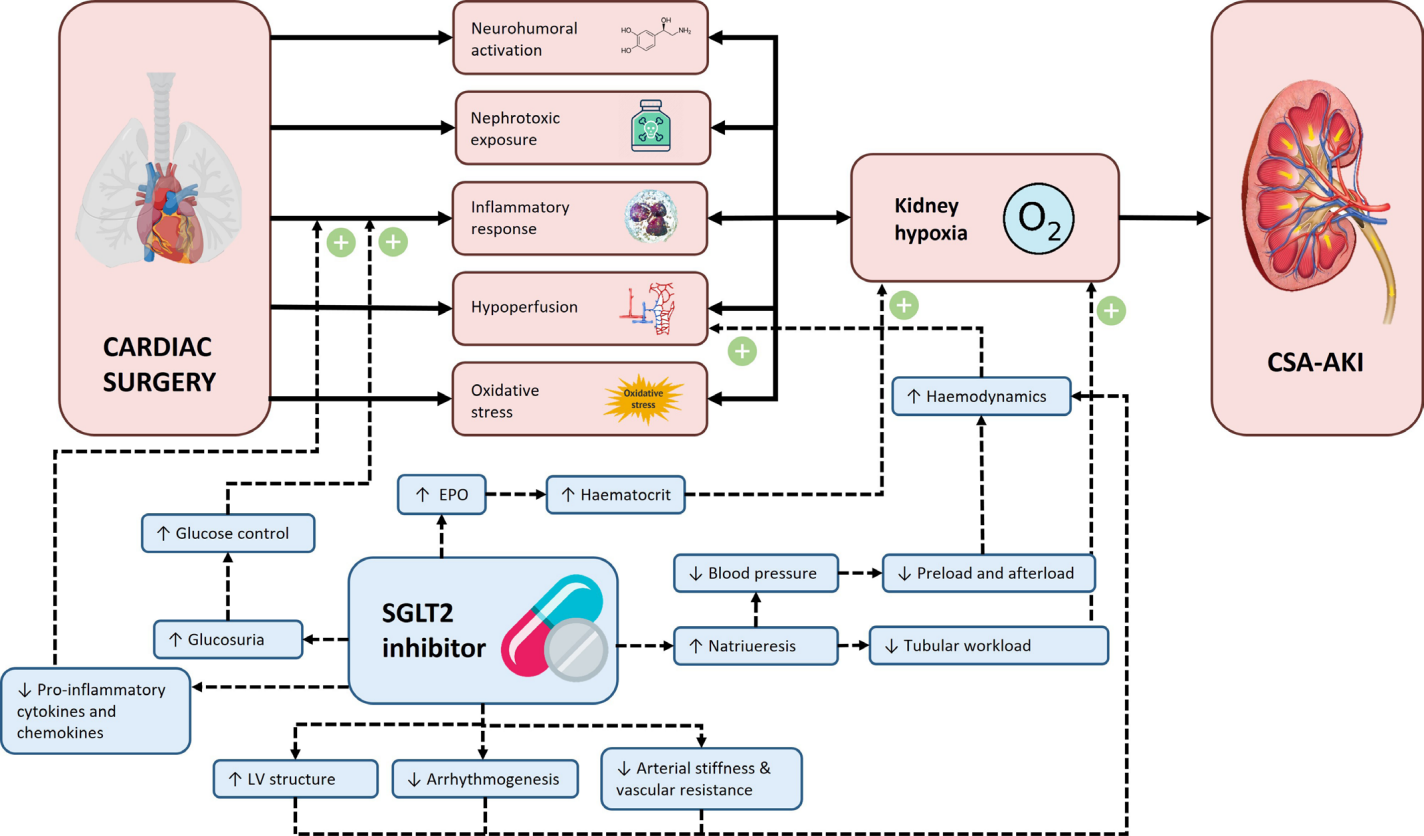
Overview of the proposed advantages of SGLT2 inhibitors in reducing CSA-AKI. CSA-AKI, cardiac surgery-associated acute kidney injury; EPO, erythropoietin; LV, left ventricle; SGTL2, sodium-glucose cotransporter-2.

Despite several attempts with a variety of pharmacological interventions, including vasodilators, diuretics, analgesics, antioxidants, cholesterol-lowering and anti-inflammatory drugs, no successful interventions have been identified that prevent CSA-AKI.^7 8^ However, sodium-glucose cotransporter-2 (SGLT2) inhibitors are promising new drugs to prevent CSA-AKI. SGLT2 inhibitors were initially developed for their glucose-lowering effects by inducing glucosuria.^4^ In addition, they have obtained a prominent place in clinical practice as they reduced progression of kidney disease in patients with CKD with or without type 2 diabetes (T2D) in large outcome trials.^9^ Moreover, in these trials, SGLT2 inhibitors reduced AKI incidence (HR 0.66, 95% CI 0.54 to 0.80), even though it was assessed as a safety endpoint in most trials.^6^ The mechanisms by which SGLT2 inhibitors prevent acute or chronic kidney injury are still the subject of research. SGLT2 inhibitors reduce estimated glomerular filtration rate and glomerular pressure, thereby preventing kidney damage.^10^ Lowering glomerular hyperfiltration reduces kidney oxygen consumption and may thus ameliorate kidney hypoxia, and since oxygen deprivation is a major contributor to CSA-AKI, this might protect against injury.^11^ Furthermore, SGLT2 inhibitors improve haemodynamics and exhibit anti-inflammatory properties.^12 13^ Preclinical data have shown that SGLT2 inhibitors reduced renal ischaemia-reperfusion injury.^14^,^16^ An overview of the proposed advantages of SGLT2 inhibitors in reducing CSA-AKI is shown in figure 1.

We conducted a pilot trial to test the feasibility and kidney protection of SGLT2 inhibitors in the context of cardiac surgery, the Metabolic and Renal outcomes in Cardiac sUrgery patients Receiving SGLT2 Inhibitors (MERCURI), at the Amsterdam University Medical Centre (UMC). This single-centre, open label, randomised, phase IV clinical trial included 55 patients and showed a significant reduction in AKI incidence of 46.7% (95% CI −69.7 to −23.6, p=0.001), comparing the use of an SGLT2 inhibitor (empagliflozin) with the untreated control group.^17^

The current MERCURI-2 trial investigates the effect of perioperative SGLT2 inhibition on the incidence of AKI in patients undergoing cardiac surgery.

## Methods

The MERCURI-2 trial (clinicaltrials.gov identifier: NCT05590143) is a multicentre randomised triple-blind placebo-controlled (1:1) superiority trial in patients undergoing cardiac surgery. The trial is designed to evaluate the effect of dapagliflozin 10 mg once daily, compared with placebo, added to standard of care, on the incidence of CSA-AKI. This is an investigator-initiated study, with Amsterdam UMC as sponsor. The study protocol was approved by the Medical Ethics Committee of the Amsterdam UMC and by the Competent Authorities according to national and international regulations (METC 2022.0795 and EudraCT number 2022-002453-25). The first patient was included in June 2023. The trial has an estimated duration of 3 years.

### Trial population

Our aim is to enrol 784 adult patients (aged above 18 years) scheduled to undergo elective cardiac surgery. Detailed exclusion criteria are listed (table 1). Patients with type 1 diabetes (T1D) are excluded because of the risk of SGLT-2 inhibitor-associated euglycaemic diabetic ketoacidosis (euDKA) in this patient population. For the same reason, patients with a history of DKA are excluded. Furthermore, patients with T2D with body mass index (BMI) <25 kg/m^2^ using multiple daily insulin injections are excluded as they phenotypically resemble T1D, placing them at higher risk for euDKA. Patients with a systolic blood pressure below 100 mm Hg at the time of inclusion are excluded due to the possible blood pressure lowering characteristics of dapagliflozin. Furthermore, patients currently treated with SGLT2 inhibitors or with known or suspected allergy to dapagliflozin are excluded from participation.

**Table 1:** Inclusion and exclusion criteria of the MERCURI-2 trial

Inclusion criteria	Exclusion criteria
Signed informed consent	T1D
Aged above 18 years	T2D with a BMI <25 kg/m^2^ and using multiple daily insulin injections (both short-acting and long-acting insulin)
Scheduled for elective cardiac surgery	History of diabetic ketoacidosis
	Systolic blood pressure <100 mm Hg at time of inclusion
	Reduced kidney function at baseline with eGFR <20 mL/min at time of inclusion
	Current treatment with SGLT2 inhibitors
	Known or suspected allergy to trial products or other drugs in the same class
	Emergency surgery, defined as in need of surgery for medical reasons within 72 hours
	Woman of childbearing potential who is pregnant, breast feeding or intends to become pregnant or is not using adequate contraceptive methods

BMI, body mass index; eGFR, estimated glomerular filtration rate; SGTL2, sodium-glucose cotransporter-2; T1D, type 1 diabetes; T2D, type 2 diabetes.

### Study design

**Figure 2:**
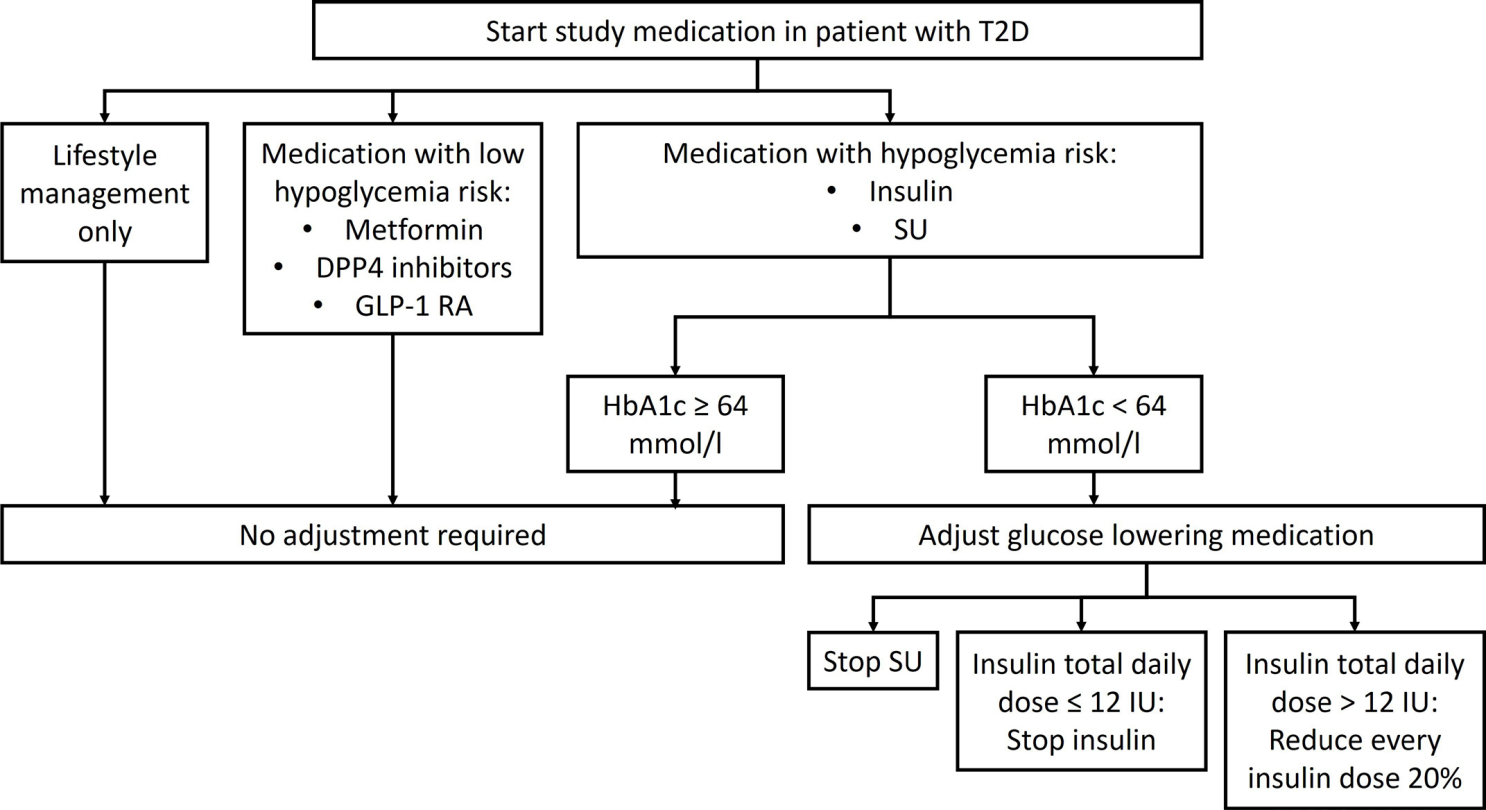
Treatment algorithm for adjustment of glucose lowering therapy in patients with T2D. DPP4, dipeptidyl peptidase IV; GLP-1 RA, glucagon like peptide-1 receptor agonist; HbA1c, haemoglobin A1c; IU, international unit; SU, sulfonylureas; T2D, type 2 diabetes.

Potentially eligible patients who are scheduled for cardiac surgery will be contacted via telephone, during preoperative hospital admission or during a preoperative hospital visit. Patients will receive both written and oral information (online supplemental file 1). After informed consent is obtained and signed, baseline characteristics such as age, sex, ethnicity, BMI, blood pressure, American Society of Anesthesiologist physical status, medical history and medication use will be collected. The most recent serum creatinine and glycated haemoglobin A1c, if measured as part of standard medical care before surgery, will be recorded. Patients will subsequently be randomised to dapagliflozin or placebo. Patients will take the first dapagliflozin 10 mg or placebo on the day before surgery, between 15.00 and 20.00. The second dosage will be taken on the morning before surgery. The remainder dosages will be taken the first and second day after surgery. In case the surgery is postponed, an additional dose will be administered. Patients with T2D on glucose lowering therapy will be advised on how to adjust their current diabetes treatment to prevent potential hypoglycaemia (figure 2).

**Figure 3:**
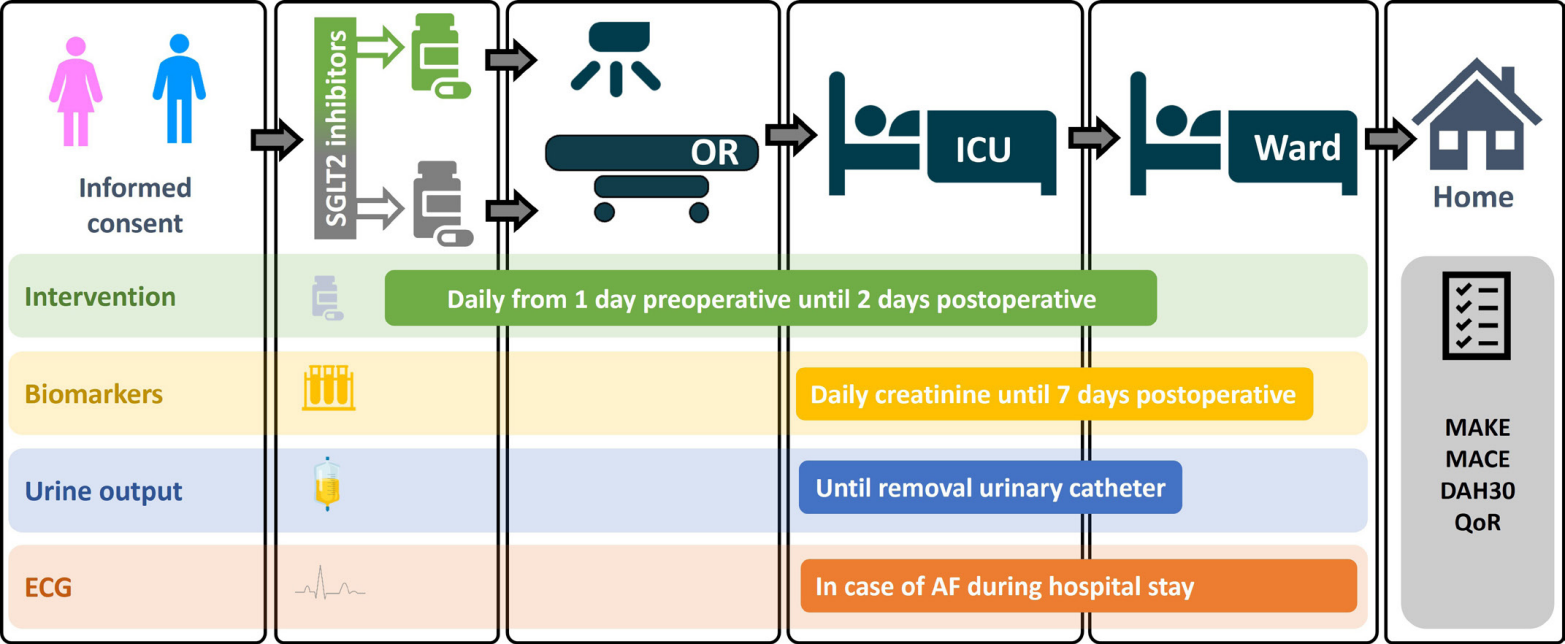
Summary of the study flow of the MERCURI-2 trial. AF, atrial fibrillation; DAH30, days at home in first 30 days; ICU, intensive care unit; MACE, major adverse cardiovascular events; MAKE, major adverse kidney events; OR, operation room; QoRquality of recovery; SGLT2, sodium-glucose cotransporter-2.

Serum creatinine will be measured daily until day 7 or until discharge from hospital. We aim to collect an additional blood sample from 20% of the participants, both on the day before and after surgery, for potential future analyses. Urine output will be reported as measured during routine medical care, until the urinary catheter is removed. Complications (online supplemental table 1) and ICU and hospital length of stay will be registered until 30 days after surgery. After 30 days, patients will receive a questionnaire measuring patient-reported quality of life and disability with questions from the WHO Disability Assessment Schedule 2.0 (WHO-DAS 2.0), Days at Home in first 30 days (DAH30) and 5-level EuroQol 5-dimensions (EQ5D5L). Data will be collected using Castor EDC (Amsterdam, Netherlands), a good clinical practice compliant data management system.^18^ The study design is summarised in figure 3.

### Randomisation

After inclusion, patients will be randomised by Castor EDC. Block randomisation with computer-generated blocks of 4, 6 or 8 patients will be used. The block size will be unknown to the researchers. Randomisation is stratified for sex, presence of T2D and study site.

### Allocation concealment and blinding

Investigators will register the patient in Castor EDC. The randomisation outcome will be exclusively visible to the trial pharmacy. Treatment allocation of a patient will only be disclosed in case of a suspected unexpected serious adverse reaction (SUSAR). Study treatment is provided in identical boxes, each containing either six overencapsulated tablets of dapagliflozin or placebo. To ensure blinding, the original 10 mg dapagliflozin (AstraZeneca, Södertälje, Sweden) tablets will be overencapsulated by the good manufacturing practice (GMP) certified trial pharmacy at Amsterdam UMC. The capsules will be filled with microcrystalline cellulose PH102. The matching placebo capsules will solely contain microcrystalline cellulose PH102. Patients, healthcare providers and investigators will be blinded to group allocation until database lock.

### Outcomes and outcome adjudication

The primary outcome of this study is the incidence of AKI according to the KDIGO criteria (table 2) based on serum creatinine and urine output. Serum creatinine levels and urine output will be reviewed by a second investigator and the study monitor to ensure primary outcome adjudication. Other secondary kidney-related outcome parameters include the difference in the primary outcome between men and women, incidence of individual AKI stages according to KDIGO criteria, the maximum change of creatinine postoperatively compared with the baseline creatinine preoperatively, the postoperative creatinine course, the number of patients with persistent kidney failure (defined as creatinine concentration ≥200% of baseline creatinine concentration after 30 days), the number of patients requiring dialysis and the number of patients who died due to a primary kidney-related cause. The cardiac-related secondary outcomes include the incidence of de novo postoperative atrial fibrillation, registered on a 12-lead ECG, the maximum postoperative creatine kinasemyocardial band (CK-MB) or troponin concentrations, the number of patients who developed new onset cardiac arrhythmia, new myocardial infarctions or cerebrovascular accident confirmed by CT scan within the first 30 days postoperatively, as well as the number of patients who required an extended hospital stay due to heart failure, needed readmission for heart failure or died from a primary cardiac cause. Furthermore, length of stay in the ICU and the hospital measured in days and all-cause mortality were collected (table 3). Complications within the first 30 days after surgery (online supplemental table 1) are retrieved from the electronic patient file and collected. The outcomes of the patient-reported quality of life and disability measured with three questionnaires, DAH30,^19^ WHO-DAS 2.0^20^ and EQ5D5L,^21^ will be evaluated across the treatment groups. Safety outcomes of SGLT2 inhibitors such as genital mycotic infections, DKA and hypoglycaemia will be assembled and compared between the treatment groups.

**Table 2:** Definition and staging of acute kidney injury according to kidney disease: improving global outcomes criteria

Stage	Acute kidney injury	Difference	Time frame
1	Serum creatinine	≥0.3 mg/dL (26.5 mcmol/l) or	Within 48 hours
		≥1.5 times baseline	Within 7 days
	Urine output	<0.5 mL/kg/h	For 6–12 hours
2	Serum creatinine	2.0–2.9 times baseline	
	Urine output	<0.5 mL/kg/h	For >12 hours
3	Start renal replacement therapy		
	Serum creatinine	3.0 times baseline or	Within 7 days
		≥4.0 mg/dL	
	Urine output	<0.3 mL/kg/h or	>24 hours
		Anuria	>12 hours

**Table 3:** Recorded secondary and exploratory outcomes

Outcome measures	Time frame	Definition
Secondary outcomes		
Individual AKI stages	≤ 7 days postoperative	Incidence of stage 1, 2 and 3 AKI according to KDIGO criteria
Creatinine	≤ 7 days postoperative	Postoperative maximum change of creatinine compared with baseline creatinine
AF	≤ 7 days postoperative	Postoperative AF recorded with an ECG or for which treatment is initiated
LoS-ICU	≤ 30 days postoperative	LoS-ICU, measured in days from transfer to ICU until discharge from ICU
LoS-Hos	≤ 30 days postoperative	LoS-Hos, measured in days from surgery until discharge from the hospital
MAKE	≤ 30 days postoperative	MAKE. Composite endpoint of death, new dialysis and worsened renal function
MACE	≤ 30 days postoperative	MACE. Composite endpoint of cardiovascular death, nonfatal myocardial infarction, nonfatal ischaemic cerebral vascular accident and hospitalisation for heart failure
Safety outcomes	≤ 30 days postoperative	Genital mycotic infections, diabetic ketoacidosis and hypoglycaemia
Hypoglycaemia	≤ 3 days postoperative	Incidence of hypoglycaemia (blood glucose <4 mmol/L) detected during routine perioperative glucose measurements
Hyperglycaemia	≤ 3 days postoperative	Incidence of hyperglycaemia (blood glucose >10 mmol/L) detected during routine perioperative glucose measurements
Cardiac biomarker 1: troponin	From transfer to ICU until 48 hours postoperatively	Peak troponin concentration as routinely measured during clinical practice
Cardiac biomarker 2: CK-MB	From transfer to ICU until 48 hours postoperatively	Peak CK-MB concentration as routinely measured during clinical practice
Patient-reported quality of recovery 1	Recorded at 30 days postoperatively	According to Days at Home in first 30 days
Patient-reported quality of recovery 2	Recorded at 30 days postoperatively	According to WHO Disability Assessment Schedule 2.0
Patient-reported quality of recovery 3	Recorded at 30 days postoperatively	According to 5 level EuroQol 5-Dimensions questionnaire
Exploratory outcomes		
Systemic haemodynamics 1	From start of anaesthesia until discharge from the ICU, assessed up to 72 hours	Perioperative hourly average heart rate
Systemic haemodynamics 2	From start of anaesthesia until discharge from the ICU, assessed up to 72 hours	Perioperative hourly average mean arterial blood pressure
Systemic haemodynamics 3	From start of anaesthesia until discharge from the ICU, assessed up to 72 hours	Perioperative hourly average cardiac output (L/min)
Urinary oxygenation	From start of anaesthesia until discharge from the ICU, assessed up to 72 hours	Perioperative hourly average mean oxygen tension measured in the bladder
Postoperative LVF	≤ 30 days postoperative	Qualitative assessment (categorised as normal or mildly, moderately or severely reduced function) of LVF as noted by the echocardiographer for routinely performed postoperative echocardiography performed during routine follow-up
Healthcare costs	Recorded at 30 days postoperatively	Using the iMCQ: IMTA (Institute for Medical Technology Assessment) Medical Consumption Questionnaire
Productivity costs	Recorded at 30 days postoperatively	Using the iPCQ: IMTA (Institute for Medical Technology Assessment) Productivity Cost Questionnaire

AF, atrial fibrillation; AKI, acute kidney injury; CK-MB, Creatine kinase myocardial band; ICU, intensive care unit; KDIGO criteria, kidney disease improving global outcomes; LoS-Hos, length of stay in the hospital; LoS-ICU, length of stay in the intensive care unit; LVF, left ventricle function; MACE, major adverse cardiovascular events; MAKE, major adverse kidney events.

### Study oversight and organisation

The core team of the MERCURI-2 investigator group consists of three principal investigators from the Amsterdam UMC and the trial coordinator. This team is responsible for the design of the study protocol and progress of the trial. The core team will draft the final report that will be approved by all MERCURI-2 investigators. The trial is conducted under supervision of an independent Data and Safety Monitoring Board (DSMB), consisting of two clinical experts and an epidemiologist. An independent statistician will present unblinded data to the DSMB. The DSMB reviews the incidence of AKI in the placebo group after 25% of the participants have been included, in order to inform the sample size calculation. The DSMB reviews the incidence of AKI at two predefined intervals (after inclusion of 25% and 50% of participants) to assess safety outcomes including AKI. No specific stop criteria were determined.

The study will be monitored by the Clinical Research Unit from the Amsterdam UMC. Each involved study site will receive a baseline and close-out visit and additional visits after the inclusion of 10 and 100 participants during the study. The monitor will approve informed consent papers, verify the serious adverse events (SAEs) and check data collection and quality of registration.

Regarding SAEs, the sponsor will yearly present an overview of the SAEs via Clinical Trials Information System (CTIS). SUSARs will be reported earlier via EudraVigilance depending on the seriousness of the reaction and will be as follows: in the case of fatal or life-threatening SUSARs, as soon as possible and in any event not later than 7 days after the sponsor became aware of the reaction; in the case of non-fatal or non-life-threatening SUSARs, not later than 15 days after the sponsor became aware of the reaction and in the case of SUSARs which were initially considered to be non-fatal or non-life-threatening but which turn out to be fatal or life-threatening. as soon as possible and in any event not later than 7 days after the sponsor became aware of the reaction being fatal or life-threatening.

### Sample size calculation

Sample size is based on Fisher’s exact test, with an expected AKI incidence in the placebo group of 22%, based on previous cohorts,^2^ taking a conservative estimation. A relative risk of 0.64 based on AKI reduction by SGLT2 inhibitors in previous trials^6^ translates into an absolute risk reduction of 7.9% and incidence in the intervention group of 14.1%. The required total sample size to find such a difference with two-sided alpha of 0.05 and 80% power is 784. We therefore aim to include 392 patients per arm.

### Achieve sample size

Based on previous trials and the short trial duration per patient, we expect a low number of drop-outs. If a drop-out occurs, for example due to surgery cancellation or withdrawal of informed consent, dropouts will be replaced to maintain sufficient statistical power.

#### Statistical analyses

The baseline characteristics of all subjects, per treatment group, will be outlined in a table describing variables such as demographic variables, weight, length, relevant medical history and current medication use. Continuous data will be presented as mean with SD or as median with IQR, depending on the distribution of the data. Distribution of data will be assessed with histograms, Q-Q plots and the Shapiro-Wilk test. We expect a small percentage of missing data due to the in-hospital setting and short duration of the study.

### Primary study outcome

Statistical analyses will be based on an intention-to-treat approach. The main analysis will concern the comparison of the incidence of AKI between both groups. The disparity in AKI incidence will be evaluated using the Fisher’s exact test. In addition, the risk difference of AKI between both groups, along with the associated 95% CIs, will be presented.

### Secondary study outcomes

Heterogeneity of treatment effect between men and women will be studied for the primary outcome by examining a treatment-by-sex interaction effect in a logistic regression model with treatment and sex as main effects. Similarly, the heterogeneity of treatment effect in patients with and without T2D will be studied using the same approach. Treatment effect estimates, along with their corresponding 95% CIs, will be reported for each specific subgroup. Other secondary outcomes will be presented as number of events and analysed with the Fisher’s exact test. Continuous data will be presented as mean with SD and differences will be assessed employing the Student’s t-test. Generalised mixed-effect models will be constructed to examine potential differences in the repeated measurements. Predefined post hoc analysis with regression analyses will be conducted to estimate the influence of age, BMI, baseline kidney function and the presence of heart failure on the treatment effect and their possible interactions on the primary outcome. A 2-sided p value <0.05 will be considered statistically significant. Statistical uncertainty will be expressed in 2-sided 95% CIs.

## Ethics and dissemination

All participants will receive a written patient information folder, accompanied by additional oral explanation from the study personnel. A subject screening and enrolment log will only be accessible to study personnel and saved on a secure server. Whenever a patient is enrolled in the study, participation in the trial will be recorded in the electronic patient database, visible for all involved healthcare workers. A subjects’ insurance has been taken out. The study protocol was approved by the Medical Ethics Committee of the Amsterdam UMC (METC 2022.0795). After completion of the trial, results will be disseminated to participants, patient societies and physicians via a network meeting and digital newsletter. Results will be submitted for publication in a peer-reviewed international medical journal and presented on (inter)national congresses.

### Planning

The first patient was included in June 2023. The trial has an estimated duration of 3 years. Whenever amendments to the protocol will be made, they will be subjected to the Ethics Committee of the Amsterdam UMC for approval and subsequently communicated to all involved study personnel. As sponsor, the Amsterdam UMC will remain owner of all data and rights to publication. The manuscript will be drafted by the core team and approved by the MERCURI-2 investigators.

### Data statement

Full protocol, dataset and data management plan will be available upon reasonable request.

## Supplementary material

10.1136/bmjopen-2024-095504 online supplemental file 1

10.1136/bmjopen-2024-095504 online supplemental file 2
