# Correction: Study protocol of the multicentre, randomised, triple-blind, placebo-controlled MERCURI-2 trial: promoting effective renoprotection in cardiac surgery patients by inhibition of sodium glucose cotransporter (SGLT)-2

**DOI:** 10.1136/bmjopen-2024-095504corr1

**Published:** 2025-08-16

 Oosterom-Eijmael M, Monteiro de Oliveira NP, Niesten ED, *et al* Study protocol of the multicentre, randomised, triple-blind, placebo-controlled MERCURI-2 trial: promoting effective renoprotection in cardiac surgery patients by inhibition of sodium glucose cotransporter (SGLT)−2. *BMJ Open* 2025;15:e095504. doi: 10.1136/bmjopen-2024–0 95 504

The author has identified errors that need to be corrected for clarity and accuracy:

In the section ‘Methods - Study oversight and organisation’, the manuscript incorrectly stated that efficacy outcomes were assessed:

‘The trial is conducted under supervision of an independent Data and Safety Monitoring Board (DSMB), consisting of two clinical experts and an epidemiologist. An independent statistician will present unblinded data to the DSMB. The DSMB reviews the incidence of AKI at two predefined intervals (after inclusion of 200 and 400 participants) to assess safety and efficacy outcomes. The DSMB can advise to stop the trial if patient safety is at risk or in case of overwhelming efficacy.’

The correct description should read:

‘The trial is conducted under supervision of an independent Data and Safety Monitoring Board (DSMB), consisting of two clinical experts and an epidemiologist. An independent statistician will present unblinded data to the DSMB. The DSMB reviews the incidence of AKI in the placebo group after 25% of the participants have been included, in order to inform the sample size calculation. The DSMB reviews the incidence of AKI at two predefined intervals (after inclusion of 25% and 50% of participants) to assess safety outcomes including AKI. No specific stop criteria were determined.’

