# Supplementary material for: Study protocol of the multicentre, randomised, triple-blind, placebo-controlled MERCURI-2 trial: promoting effective renoprotection in cardiac surgery patients by inhibition of sodium glucose cotransporter (SGLT)-2
Source: BMJ Open. 2025 May 16;15(5):e095504. doi: 10.1136/bmjopen-2024-095504 (PMC12086897; doi:10.1136/bmjopen-2024-095504)
Supplement: online supplemental file 1 [file bmjopen-15-5-s001.docx]

**Proefpersoneninformatie voor deelname
aan medisch-wetenschappelijk onderzoek**

**Dapagliflozine ter vermindering van acute nierschade na een hartoperatie - De MERCURI-2 studie**

*proMoting Effective Renoprotection in Cardiac sURgery patients by SGLT2- Inhibitors*

**Inleiding**

Geachte heer/mevrouw,

Met deze informatiebrief willen we u vragen of u wilt meedoen aan medisch-wetenschappelijk onderzoek. Meedoen is vrijwillig. U krijgt deze brief omdat u binnenkort een hartoperatie ondergaat. U leest hier om wat voor onderzoek het gaat, wat het voor u betekent, en wat de voordelen en nadelen zijn. Het is veel informatie. Wilt u de informatie doorlezen en beslissen of u wilt meedoen? Als u wilt meedoen, kunt u het formulier invullen dat u vindt in bijlage D.

***Stel uw vragen***

U kunt uw beslissing nemen met de informatie die u in deze informatiebrief vindt. Daarnaast raden we u aan om dit te doen:

- Stel vragen aan de onderzoeker die u deze informatie geeft.

- Praat met uw partner, familie of vrienden over dit onderzoek.

- Stel vragen aan de onafhankelijk deskundige. Voor contactgegevens zie bijlage A

- Lees de informatie op [www.rijksoverheid.nl/mensenonderzoek](http://www.rijksoverheid.nl/mensenonderzoek).

1. **Algemene informatie**

AMC heeft dit onderzoek opgezet. Hieronder noemen we het AMC steeds de ‘opdrachtgever’. Onderzoekers, dit kunnen ook artsen en verpleegkundigen zijn, voeren het onderzoek uit in verschillende ziekenhuizen.

Deelnemers aan een medisch-wetenschappelijk onderzoek worden vaak proefpersonen genoemd. Zowel patiënten als mensen die gezond zijn, kunnen proefpersoon zijn.

In Nederland zullen naar verwachting 784 meedoen.

De medisch-ethische toetsingscommissie van het AMC heeft dit onderzoek goedgekeurd.

**2. Wat is het doel van het onderzoek?**

In dit onderzoek bekijken we hoe veilig het nieuwe middel dapagliflozine is voor de behandeling van acuut nierfalen. En hoe goed het werkt.

We vergelijken de werking van dapagliflozine met de werking van een placebo. Een placebo is een middel zonder werkzame stof, een ‘nepmiddel’.

1. **Wat is de achtergrond van het onderzoek?**

Veel patiënten hebben tijdelijk een verminderde nierfunctie na een hartoperatie. Dit komt door schadelijke stoffen die in het bloed komen tijdens een hartoperatie. Zowel door het snijden van de chirurg, de verstoring van de bloedsomloop alsook de hartlongmachine die wordt gebruikt. In de meeste gevallen herstelt de nierfunctie in de dagen na de operatie, maar voor een kleine groep duurt deze verstoring langer en sommigen hebben zelfs een nierfunctie vervangende therapie (dialyse) nodig.

In deze studie onderzoeken wij een middel dat mogelijk de nieren kan beschermen. Dapagliflozine is een middel dat al jaren veel wordt gebruikt in de behandeling van mensen met suikerziekte (diabetes mellitus). Deze ziekte veroorzaakt nierschade. Dit middel verhoogt de uitscheiding van suiker en natrium (zout) door de nieren. Uit eerder onderzoek blijkt dat bij mensen met suikerziekte die dit middel krijgen minder nierschade optreed. In ons onderzoek bekijken we of dapagliflozine ook een beschermend effect heeft op de nieren in mensen die een hartoperatie ondergaan. Dit willen we testen in zowel mensen met als zonder suikerziekte. Vervolgens vergelijken we een groep die dapagliflozine neemt in de periode voor en na hun operatie met een groep die een placebo (nepmiddel) neemt. We denken dat de mensen die dapagliflozine nemen minder vaak nierschade ontwikkelen na hun operatie.

1. **Hoe verloopt het onderzoek?**

*Hoelang duurt het onderzoek?*

Doet u mee met het onderzoek? Dan duurt dat in totaal ongeveer 1 maand.

*Stap 1: bent u geschikt om mee te doen?*

We willen eerst weten of u geschikt bent om mee te doen. Daarom stelt de onderzoeker een aantal vragen en bekijkt uw medisch dossier. De onderzoeker zal ook vragen naar uw etnische afkomst, omdat het risico op nierschade verschillend is voor mensen met verschillende genetische achtergronden.

*Stap 2: de behandeling*

We behandelen u 4 dagen met onderzoeksmiddelen.

Voor dit onderzoek maken we 2 groepen:

- Groep 1. De mensen in deze groep krijgen dapagliflozine.
- Groep 2. De mensen in deze groep krijgen placebo.

Loting bepaalt welke behandeling u krijgt. U en de onderzoeker weten niet in welke groep u zit. Als het voor uw gezondheid belangrijk is, kan dit wel worden opgezocht.

*Stap 3: onderzoeken en metingen*

Voor het onderzoek is het niet nodig dat u extra naar het ziekenhuis komt. Het onderzoek vindt plaats tijdens uw opname in het ziekenhuis. Dertig dagen na uw operatie bellen of e-mailen we nog een keer met een vragenlijst over uw ervaringen na de operatie.

We doen de volgende onderzoeken:

- Onderzoek van uw bloed. Daarvoor neemt de onderzoeker per keer 1 buisje bloed af. Alles bij elkaar nemen we 12 ml bloed bij u af. Met het bloedonderzoek testen we uw nierfunctie voor de en na de operatie. Verder gebruiken we de uitslagen van bloedonderzoek dat tijdens uw gewone zorg wordt afgenomen.
- Voor de operatie begint krijgt u standaard een katheter om de urine af te laten lopen tijdens en de eerste tijd na de operatie. Voor het onderzoek meten we met een extra sensor de zuurstofspanning in de urine.
- U vult een vragenlijst in. De vragen gaan over de kwaliteit van herstel na uw operatie en vraagt of er complicaties zijn opgetreden. Deze vragenlijst kunt u online invullen en kunt u via email toegestuurd krijgen invullen kost u ongeveer 30 minuten. Als u dat liever heeft, kan de onderzoeker u ook 1 keer opbellen. U krijgt dan de vragen uit de vragenlijst mondeling gesteld. Dit telefoongesprek duurt tevens ongeveer 30 minuten.

*Wat is er anders dan bij gewone zorg?*

Er is bij dit onderzoek niet zoveel anders dan bij gewone zorg. Deelname aan dit onderzoek heeft verder geen invloed op de gewone zorg en de operatie die u ondergaat, en zal bijvoorbeeld niet tot vertraging of uitstel leiden.

1. **Welke afspraken maken we met u?**

We willen graag dat het onderzoek goed verloopt. Daarom maken we de volgende afspraken met u

- U neemt het medicijn op de manier die de onderzoeker u heeft uitgelegd.
- U doet tijdens dit onderzoek niet mee aan een ander medisch-wetenschappelijk onderzoek.
- U neemt contact op met de onderzoeker in deze situaties:
  - U wilt andere medicijnen gaan gebruiken. Ook als dit homeopathische middelen, natuurgeneesmiddelen, vitaminen of geneesmiddelen van de drogist zijn.
  - U wordt in een ziekenhuis opgenomen of behandeld.
  - U krijgt plotseling problemen met uw gezondheid.
  - U wilt niet meer meedoen met het onderzoek.
  - Uw telefoonnummer of e-mailadres verandert.

*Mag u of uw partner zwanger worden tijdens het onderzoek?*

Vrouwen die zwanger zijn of borstvoeding geven, kunnen niet meedoen aan dit onderzoek. Vrouwen mogen ook niet zwanger worden tijdens het onderzoek.

Dit onderzoek kan namelijk gevolgen hebben voor een ongeboren kind. De onderzoeker vertelt u hoe u het beste een zwangerschap voorkomt. Praat hierover met uw partner.

*Toch zwanger?*

Wordt u toch zwanger tijdens het onderzoek? Laat dit dan meteen weten aan de onderzoeker. U moet dan in overleg met de onderzoeker zo snel mogelijk stoppen met dit onderzoek. Wordt uw partner zwanger van u tijdens het onderzoek? Vraag haar dan toestemming om dit aan de onderzoeker te laten weten. Dan kan de zwangerschap extra gecontroleerd worden en kan informatie over het verloop en de uitkomst van de zwangerschap bij andere hulpverleners worden opgevraagd. Maar alleen als u/ uw zwangere partner daar toestemming voor geeft.‘*Zwanger worden na het onderzoek*?’

De onderzoeker zal met u bespreken of deelname aan het onderzoek gevolgen kan hebben als u later zwanger wilt worden.

1. **Van welke bijwerkingen kunt u last krijgen?**

Dapagliflozine kan bijwerkingen geven. Bijwerkingen zijn zeldzaam en in principe mild en goed te behandelen.

De volgende bijwerkingen komen soms voor:

- - Dorst, duizeligheid, lage bloeddruk.
  - Urineweginfecties
  - Lager bloedsuiker als u andere bloedsuikerverlagende therapie gebruikt.

Het onderzoeksmiddel kan ook bijwerkingen hebben die nog onbekend zijn, als deze aan het licht komen tijdens de looptijd van deze studie stellen wij u hiervan op de hoogte.

Meer informatie over dapagliflozine staat in de bijsluiter. Doet u mee aan het onderzoek? Dan krijgt u de bijsluiter mee bij het middel.

*Wat zijn de mogelijke ongemakken van metingen tijdens het onderzoek?*

Bloedafname kan wat pijn doen. Of u kunt daardoor een bloeduitstorting krijgen. De meeste bloedafnames zullen we echter doen uit het infuus dat u toch al krijgt tijdens uw opname. U zult daarom zo min mogelijk geprikt worden voor dit onderzoek.

1. **Wat zijn de voordelen en de nadelen als u meedoet aan het onderzoek?**

Meedoen aan het onderzoek kan voordelen en nadelen hebben. Hieronder zetten we ze op een rij. Denk hier goed over na, en praat erover met anderen.

Dapagliflozine kan het waarschijnlijk risico op nierfalen na uw operatie verminderen, maar zeker is dat nog niet. Daarom doen we dit onderzoek.

Meedoen aan het onderzoek kan deze nadelen of gevolgen hebben:

- - U kunt last krijgen van de bijwerkingen of nadelige effecten van dapagliflozine. U kunt last hebben van de metingen tijdens het onderzoek.
  - Meedoen aan het onderzoek kost u extra tijd.
  - U moet zich houden aan de afspraken die horen bij het onderzoek.

*Wilt u niet meedoen?*

U beslist zelf of u meedoet aan het onderzoek. Wilt u niet meedoen? Dan krijgt u de gewone behandeling indien toch acuut nierfalen optreedt, meestal betekent dit afwachten of het overgaat, en als deze ernstig is nierfunctie vervangende therapie door middel van een dialyse machine. Uw arts kan u meer vertellen over de behandelingsmogelijkheden die er zijn. En over de voor- en nadelen daarvan.

1. **Wanneer stopt het onderzoek?**

De onderzoeker laat het u weten als er nieuwe informatie over het onderzoek komt die belangrijk voor u is. De onderzoeker vraagt u daarna of u blijft meedoen.

In deze situaties stopt voor u het onderzoek:

- Alle onderzoeken volgens het schema zijn voorbij.
- U bent zwanger geworden.
- U wilt zelf stoppen met het onderzoek. Dat mag op ieder moment. Meld dit dan meteen bij de onderzoeker. U hoeft er niet bij te vertellen waarom u stopt. U krijgt dan weer de gewone behandeling en operatie. De onderzoeker zal u nog wel uitnodigen voor een nacontrole als u dat wenst.
- De onderzoeker vindt het beter voor u om te stoppen. De onderzoeker zal u ook dan nog wel uitnodigen voor een nacontrole.
- Een van de volgende instanties besluit dat het onderzoek moet stoppen:
  - Het AMC,
  - de overheid, of
  - de medisch-ethische commissie die het onderzoek beoordeelt.

*Wat gebeurt er als u stopt met het onderzoek?*

De onderzoekers gebruiken de gegevens en het lichaamsmateriaal zoals uw bloed die tot het moment van stoppen zijn verzameld. Als u wilt, kan verzameld lichaamsmateriaal worden vernietigd. Geef dit door aan de onderzoeker.

Het hele onderzoek is afgelopen als alle 784 deelnemers het onderzoek hebben doorlopen.

1. **Wat gebeurt er na het onderzoek?**

*Kunt u de medicatie blijven gebruiken?*

De medicatie die u tijdens dit onderzoek krijgt beschermen uw nieren tijdens een operatie. Er is dus geen reden om na de operatie deze medicatie te blijven gebruiken. Het zou wel kunnen dat u van uw huisarts of medisch specialist dezelfde medicatie krijgt voorgeschreven met een andere reden.

*Krijgt u de resultaten van het onderzoek?*

Ongeveer 12 maanden nadat het hele onderzoek is afgerond kan de onderzoeker u laten weten wat de belangrijkste uitkomsten zijn van het onderzoek. De onderzoeker kan u dan ook vertellen in welke groep u zat. Als u dit op prijs stelt kunt u dit aangeven en uw e-mailadres met ons te delen aan het einde van dit formulier.

1. **Wat doen we met uw gegevens** **en lichaamsmateriaal?**

Doet u mee met het onderzoek? Dan geeft u ook toestemming om uw gegevens en lichaamsmateriaal te verzamelen, gebruiken en bewaren.

*Welke gegevens bewaren we?*

We bewaren deze gegevens:

- uw naam

- uw geslacht

- uw geboortedatum

- uw etniciteit

- gegevens over uw gezondheid

- (medische) gegevens die we tijdens het onderzoek verzamelen

*Welk lichaamsmateriaal bewaren we?*

We verzamelen, gebruiken en bewaren buisjes bloed.

*Waarom verzamelen, gebruiken en bewaren we uw gegevens en lichaamsmateriaal?*

We verzamelen, gebruiken en bewaren uw gegevens en uw lichaamsmateriaal om de vragen van dit onderzoek te kunnen beantwoorden. En om de resultaten te kunnen publiceren. Gegevens en/of lichaamsmateriaal kunnen worden gebruikt door de opdrachtgever bij het analyseren van onderzoeksgegevens.

*Hoe beschermen we uw privacy?*

Om uw privacy te beschermen geven wij uw gegevens en uw lichaamsmateriaal een code. Op al uw gegevens en lichaamsmateriaal zetten we alleen deze code. De sleutel van de code bewaren we op een beveiligde plek in het ziekenhuis. Als we uw gegevens en lichaamsmateriaal verwerken, gebruiken we steeds alleen die code. Ook in rapporten en publicaties over het onderzoek kan niemand terughalen dat het over u ging.

*Wie kunnen uw gegevens zien?*

Sommige personen kunnen wel uw naam en andere persoonlijke gegevens zonder code inzien. Dit kunnen gegevens zijn die speciaal voor dit onderzoek zijn verzameld, maar ook gegevens uit uw medisch dossier.

Dit zijn mensen die controleren of de onderzoekers het onderzoek goed en betrouwbaar uitvoeren. Deze personen kunnen bij uw gegevens komen:

- Leden van de commissie die de veiligheid van het onderzoek in de gaten houdt.
- Een controleur die voor de opdrachtgever werkt.
- Nationale en internationale toezichthoudende autoriteiten.

Deze personen houden uw gegevens geheim. Voor inzage door deze personen vragen wij u toestemming te geven. De Inspectie Gezondheidszorg en Jeugd kan zonder uw toestemming uw gegevens inzien.

*Hoelang bewaren we uw gegevens en lichaamsmateriaal?*

We bewaren uw gegevens 25 jaar in het ziekenhuis. Uw lichaamsmateriaal bewaren we in het ziekenhuis. Het wordt 2 jaar bewaard om daarop in de loop van dit onderzoek nog nieuwe bepalingen te kunnen doen die te maken hebben met dit onderzoek. Zodra dit niet meer nodig is, vernietigen we uw lichaamsmateriaal.

*Mogen we uw gegevens en lichaamsmateriaal gebruiken voor ander onderzoek?*

Uw verzamelde gegevens en uw (overgebleven) lichaamsmateriaal kunnen ook van belang zijn voor ander wetenschappelijk onderzoek op het gebied van nierfalen na hartoperaties. Daarvoor zullen uw gegevens en lichaamsmateriaal 2 jaar worden bewaard in het ziekenhuis. In het toestemmingformulier geeft u aan of u dit goed vindt. Geeft u geen toestemming? Dan kunt u nog steeds meedoen met dit onderzoek. U krijgt dezelfde zorg.

*Kunt u uw toestemming voor het gebruik van uw gegevens weer intrekken?*

U kunt uw toestemming voor het gebruik van uw gegevens op ieder moment intrekken. Zeg dat dan tegen de onderzoeker. Dit geldt voor het gebruik in dit onderzoek en voor het gebruik in ander onderzoek. Maar let op: trekt u uw toestemming in, en hebben onderzoekers dan al gegevens verzameld voor een onderzoek? Dan mogen zij deze gegevens nog wel gebruiken. Voor uw lichaamsmateriaal geldt dat de onderzoekers dit vernietigen nadat u uw toestemming intrekt. Maar zijn er dan al metingen gedaan met uw lichaamsmateriaal? Dan mag de onderzoeker de resultaten daarvan blijven gebruiken.

*Wilt u meer weten over uw privacy?*

- Wilt u meer weten over uw rechten bij de verwerking van persoonsgegevens? Kijk dan op [www.autoriteitpersoonsgegevens.nl](http://www.autoriteitpersoonsgegevens.nl).
- Heeft u vragen over uw rechten? Of heeft u een klacht over de verwerking van uw persoonsgegevens? Neem dan contact op met degene die verantwoordelijk is voor de verwerking van uw persoonsgegevens. Voor uw onderzoek is dat: Het AMC. Zie bijlage A voor contactgegevens, en website.
- Als u klachten heeft over de verwerking van uw persoonsgegevens, raden we u aan om deze eerst te bespreken met het onderzoeksteam. U kunt ook naar de Functionaris Gegevensbescherming van het AMC gaan. Of u dient een klacht in bij de Autoriteit Persoonsgegevens.

*Waar vindt u meer informatie over het onderzoek?*

Op de volgende website(s) vindt u meer informatie over het onderzoek. [www.ClinicalTrials.gov](file:///H:\Application%20Data\Anouk\DCRF%20werkgroep%20PIF\www.ClinicalTrials.gov). Na het onderzoek kan de website een samenvatting van de resultaten van dit onderzoek tonen. U vindt het onderzoek door te zoeken op ‘NCT05590143’

1. **Krijgt u een vergoeding als u meedoet aan het onderzoek?**

De onderzoeksmiddelen, en extra testen voor het onderzoek kosten u niets. U krijgt ook geen vergoeding als u meedoet aan dit onderzoek.

1. **Bent u verzekerd tijdens het onderzoek?**

Voor iedereen die meedoet aan dit onderzoek is een verzekering afgesloten. De verzekering betaalt voor schade door het onderzoek. Maar niet voor alle schade. In **bijlage B** vindt u meer informatie over de verzekering en de uitzonderingen. Daar staat ook aan wie u schade kunt melden.

1. **We informeren uw behandelend specialist en apotheker.**

De onderzoeker stuurt uw behandelend specialist en apotheker een bericht om te laten weten dat u meedoet aan het onderzoek. Dit is voor uw eigen veiligheid.

1. **Heeft u vragen?**

Vragen over het onderzoek kunt u stellen aan de onderzoeker. Wilt u advies van iemand die er geen belang bij heeft? Ga dan naar de onafhankelijk deskundige, voor contactgegevens zie bijlage A. Hij weet veel over het onderzoek, maar werkt niet mee aan dit onderzoek.

Heeft u een klacht? Bespreek dit dan met de onderzoeker of de arts die u behandelt. Wilt u dit liever niet? Ga dan naar de klachtenfunctionaris van uw ziekenhuis. In bijlage A staat waar u die kunt vinden.

1. **Hoe geeft u toestemming voor het onderzoek?**

U kunt eerst rustig nadenken over dit onderzoek. Daarna vertelt u de onderzoeker of u de informatie begrijpt en of u wel of niet wilt meedoen. Wilt u meedoen? Dan vult u het toestemmingsformulier in dat u bij deze informatiebrief vindt. U en de onderzoeker krijgen allebei een getekende versie van deze toestemmingsverklaring.

Dank voor uw tijd.

1. **Bijlage A: contactgegevens onderzoek**

X

**Bijlage B:**

**INFORMATIE OVER DE VERZEKERING**

X

1. **Bijlage C: Schema onderzoekshandelingen**

**Vóór de opname in het ziekenhuis:**

U krijgt mondeling en schriftelijke informatie.

U tekent schriftelijk voor toestemming met deelname aan dit onderzoek.

We verzamelen enkele medische gegevens.

**Tijdens de opname voor uw operatie:**

Op de dag vóór uw operatie krijgt u de studiemedicatie. Deze neemt u voor het eerst op de dag vóór uw operatie, daarna dagelijks, in de ochtend voor de drie dagen daarna. Dat wil zeggen, 1 keer op de ochtend van uw operatie, en op de twee dagen na de operatie.

Rond uw operatie verzamelen de onderzoekers medische gegevens, wat u hier van merkt is dat er voor de operatie en in de eerste 7 dagen na uw operatie iedere dag een buisje bloed wordt afgenomen voor het meten van uw nierfunctie.

**Ná de opname voor uw operatie.**

Dertig dagen na uw operatie, sturen we u een vragenlijst, die vraagt naar uw ervaringen tijdens de opname en de weken na uw operatie. Wij vragen naar eventuele bijwerkingen, kwaliteit van herstel na de operatie en eventuele complicaties.

1. **Bijlage D: toestemmingsformulier proefpersoon**

Behorende bij

**Dapagliflozine ter vermindering van acute nierschade na een hartoperatie - De MERCURI-2 studie**

*proMoting Effective Renoprotection in Cardiac sURgery patients by SGLT2- Inhibitors*

- Ik heb de informatiebrief gelezen. Ook kon ik vragen stellen. Mijn vragen zijn goed genoeg beantwoord. Ik had genoeg tijd om te beslissen of ik meedoe.
- Ik weet dat meedoen vrijwillig is. Ook weet ik dat ik op ieder moment kan beslissen om toch niet mee te doen met het onderzoek. Of om ermee te stoppen. Ik hoef dan niet te zeggen waarom ik wil stoppen.
- Ik geef de onderzoeker toestemming om mijn behandelend medisch specialist en apotheker te laten weten dat ik meedoe aan dit onderzoek.
- Ik geef de onderzoeker toestemming om medische informatie op te vragen bij mijn huisarts en het ziekenhuis waar ik opgenomen ben vóór of ná mijn hartoperatie.
- Ik geef de onderzoeker toestemming om mijn huisarts of behandelend specialist informatie te geven over onverwachte bevindingen uit het onderzoek die van belang zijn voor mijn gezondheid.
- Ik geef de onderzoekers toestemming om mijn gegevens en lichaamsmateriaal te verzamelen en gebruiken. De onderzoekers doen dit alleen om de onderzoeksvraag van dit onderzoek te beantwoorden.
- Ik weet dat voor de controle van het onderzoek sommige mensen al mijn gegevens kunnen inzien. Die mensen staan in deze informatiebrief. Ik geef deze mensen toestemming om mijn gegevens in te zien voor deze controle.
- Ik weet dat ik niet zwanger mag worden tijdens het onderzoek.
- Indien van toepassing heeft de onderzoeker met mij besproken hoe ik het beste voorkom dat ik zwanger word.

**Wilt u in de tabel hieronder ja of nee aankruisen?**

| Ik geef toestemming om mijn gegevens te bewaren om dit te gebruiken voor ander onderzoek, zoals in de informatiebrief staat. | Ja ☐ | Nee ☐ |
| --- | --- | --- |
| Ik geef toestemming om mijn (overgebleven) lichaamsmateriaal (bloed) te bewaren om dit te gebruiken voor ander onderzoek, zoals in de informatiebrief staat. Het lichaamsmateriaal wordt daarvoor nog 2 jaar bewaard. | Ja ☐ | Nee ☐ |
| Ik geef toestemming om mij eventueel na dit onderzoek te vragen of ik wil meedoen met een vervolgonderzoek. | Ja ☐ | Nee ☐ |
| Ik geef toestemming om de zuurstofspanning in de urine te meten | Ja ☐ | Nee ☐ |
| Ik geef de onderzoekers toestemming om na het onderzoek te laten weten welke behandeling ik heb gehad/ in welke groep ik zat. | Ja ☐ | Nee ☐ |

**Ik wil meedoen aan dit onderzoek.**

Mijn naam is (proefpersoon): ……………………………………………………………..

Handtekening: ……………………………………………… Datum : __ / __ / __

Ik verklaar dat ik deze proefpersoon volledig heb geïnformeerd over het genoemde onderzoek.

Wordt er tijdens het onderzoek informatie bekend die die de toestemming van de proefpersoon kan beïnvloeden? Dan laat ik dit op tijd weten aan deze proefpersoon.

Naam onderzoeker (of diens vertegenwoordiger): …………………………………………………

Handtekening: ……………………………………………… Datum: __ / __ / __

Als u na afloop van het onderzoek graag te weten komt in welke groep u viel en de resultaten van ons onderzoek ontvangt vul dan hier uw email adres in: ………………………………………………………@………………………………

*De proefpersoon krijgt een volledige informatiebrief mee, samen met een door beide partijen getekende versie van het toestemmingsformulier.*

***English translation***

**Participant Information for Participation in Medical-Scientific Research**

**Dapagliflozin for Reducing Acute Kidney Injury After Heart Surgery – The MERCURI-2 Study***proMoting Effective Renoprotection in Cardiac sURgery patients by SGLT2 Inhibitors*

**Introduction**

Geachte heer/mevrouw,

**Dear Sir/Madam,**

With this information letter, we would like to ask if you would be willing to participate in medical-scientific research. Participation is voluntary. You are receiving this letter because you will soon be undergoing heart surgery.

In this document, you will find details about the study, what participation entails, and the potential benefits and risks. It contains a lot of information. Please take the time to read it carefully and decide whether you would like to participate. If you choose to participate, you can fill out the form found in **Appendix D**.

***Ask Questions***

You can make your decision based on the information provided in this letter. Additionally, we recommend that you:

- Ask questions to the researcher providing you with this information.
- Discuss the study with your partner, family, or friends.
- Consult an independent expert. Contact details can be found in Appendix A.
- Read more at <www.rijksoverheid.nl/mensenonderzoek>.

**General Information**

The AMC has initiated this study. Throughout this document, we will refer to AMC as the ‘sponsor.’ Researchers—including doctors and nurses—will conduct the study in various hospitals.

Participants in medical-scientific research are often referred to as study subjects. Both patients and healthy individuals can participate as study subjects.

In the Netherlands, approximately 784 participants are expected to take part in this study.

The Medical Ethics Review Committee of AMC has approved this study.

**What is the Purpose of the Study?**

This study aims to assess the safety and effectiveness of the new drug dapagliflozin for the treatment of acute kidney injury (AKI).

We will compare the effect of dapagliflozin with a placebo. A placebo is an inactive substance, often referred to as a ‘dummy drug,’ which contains no active ingredients.

**What is the Background of the Study?**

Many patients experience temporary reduced kidney function after heart surgery. This occurs due to harmful substances that enter the bloodstream during the procedure. Factors contributing to this include:

- The surgical incision,
- Disruptions in blood circulation, and
- The use of the heart-lung machine during surgery.

In most cases, kidney function recovers within a few days after surgery. However, for a small group of patients, this impairment lasts longer, and some may even require kidney replacement therapy (dialysis).

This study aims to investigate a drug that may protect the kidneys. Dapagliflozin has been widely used for years in the treatment of diabetes mellitus (diabetes). Diabetes is known to cause kidney damage. This medication increases the excretion of sugar and sodium (salt) through the kidneys.

Previous research has shown that patients with diabetes who take dapagliflozin experience less kidney damage. In this study, we aim to determine whether dapagliflozin also has a protective effect on the kidneys of patients undergoing heart surgery. We will test this in both patients with and without diabetes.

To assess this, we will compare:

- A group of patients taking dapagliflozin before and after surgery, and
- A group taking a placebo (inactive drug).

We hypothesize that patients taking dapagliflozin will be less likely to develop kidney damage after surgery.

**How is the Study Conducted?**

How Long Does the Study Last?

If you participate, the study will last approximately one month.

Step 1: Are You Eligible to Participate?

To determine your eligibility, the researcher will:

- Ask you a few questions,
- Review your medical records, and
- Inquire about your ethnic background (since the risk of kidney damage varies across genetic backgrounds).

Step 2: The Treatment

The study treatment lasts four days. Participants are divided into two groups:

- Group 1: Receives dapagliflozin
- Group 2: Receives a placebo (inactive drug)

Random assignment (lottery) determines which group you are in.
Neither you nor the researcher will know which treatment you receive. However, if it becomes medically necessary, this information can be accessed.

Step 3: Examinations and Measurements

You do not need to visit the hospital extra times for this study. The study will take place during your hospital stay. Thirty days after surgery, we will contact you via phone or email with a questionnaire about your recovery.

We will conduct the following tests:

- Blood Tests:
  - A small blood sample (1 tube) will be taken per test, totaling 12 mL.
  - These tests will evaluate your kidney function before and after surgery.
  - We will also use results from routine blood tests conducted as part of your standard care.
- Urine Tests:
  - Before surgery, a catheter will be placed (as part of standard care) to drain urine during and after the procedure.
  - During the study, an additional sensor will be used to measure oxygen levels in the urine.
- Questionnaire:
  - You will be asked about your recovery quality and whether you experienced any complications.
  - The questionnaire takes about 30 minutes to complete and can be filled out online via email.
  - If preferred, a researcher can call you to go through the questions over the phone (also around 30 minutes).

What is Different from Standard Care?

This study does not significantly alter your standard medical care.

- Participation will not affect your scheduled surgery.
- There will be no delays or changes in your treatment due to the study.

We doen de volgende onderzoeken:

- Onderzoek van uw bloed. Daarvoor neemt de onderzoeker per keer 1 buisje bloed af. Alles bij elkaar nemen we 12 ml bloed bij u af. Met het bloedonderzoek testen we uw nierfunctie voor de en na de operatie. Verder gebruiken we de uitslagen van bloedonderzoek dat tijdens uw gewone zorg wordt afgenomen.
- Voor de operatie begint krijgt u standaard een katheter om de urine af te laten lopen tijdens en de eerste tijd na de operatie. Voor het onderzoek meten we met een extra sensor de zuurstofspanning in de urine.
- U vult een vragenlijst in. De vragen gaan over de kwaliteit van herstel na uw operatie en vraagt of er complicaties zijn opgetreden. Deze vragenlijst kunt u online invullen en kunt u via email toegestuurd krijgen invullen kost u ongeveer 30 minuten. Als u dat liever heeft, kan de onderzoeker u ook 1 keer opbellen. U krijgt dan de vragen uit de vragenlijst mondeling gesteld. Dit telefoongesprek duurt tevens ongeveer 30 minuten.

*Wat is er anders dan bij gewone zorg?*

Er is bij dit onderzoek niet zoveel anders dan bij gewone zorg. Deelname aan dit onderzoek heeft verder geen invloed op de gewone zorg en de operatie die u ondergaat, en zal bijvoorbeeld niet tot vertraging of uitstel leiden.

**What Agreements Do We Make With You?**

We want the study to proceed smoothly. Therefore, we ask you to agree to the following:

- You take the medication exactly as instructed by the researcher.
- You do not participate in any other medical-scientific research during this study.
- You contact the researcher in the following situations:
  - If you plan to take other medications, including homeopathic remedies, herbal supplements, vitamins, or over-the-counter drugs.
  - If you are hospitalized or receive medical treatment.
  - If you experience sudden health problems.
  - If you wish to withdraw from the study.
  - If your phone number or email address changes.

Can You or Your Partner Become Pregnant During the Study?

Women who are pregnant or breastfeeding cannot participate in this study. Additionally, women must not become pregnant during the study, as the medication could potentially affect an unborn child. The researcher will explain how to prevent pregnancy during the study. We recommend discussing this with your partner.

What If You Become Pregnant?

If you become pregnant during the study, inform the researcher immediately. You will need to stop participation as soon as possible, in consultation with the researcher.

If your partner becomes pregnant during the study, we ask you to request her permission to inform the researcher. This will allow for additional monitoring of the pregnancy. With your partner’s consent, the researcher may also gather information from other healthcare providers about the pregnancy’s progress and outcome.

What About Pregnancy After the Study?

The researcher will discuss whether participation in the study could have any future effects on pregnancy.

**What Side Effects Might You Experience?**

Dapagliflozin can cause side effects. These are rare, usually mild, and treatable.

Some possible side effects include:

- Thirst, dizziness, low blood pressure.
- Urinary tract infections.
- Lower blood sugar levels (especially if you are taking other blood sugar-lowering medications).

There may also be unknown side effects that are discovered during this study. If any new side effects become known, we will inform you.

For more information about dapagliflozin, please refer to the medication leaflet. If you participate, you will receive this leaflet along with the medication.

What Are the Possible Discomforts From the Study Measurements?

- Blood draws may cause mild pain or bruising.
- However, most blood samples will be taken from the IV line already placed during your hospital stay, so you will be pricked as little as possible.

**What Are the Benefits and Risks of Participating in the Study?**

Participating in this study may have both benefits and drawbacks. Please consider these carefully and discuss them with others.

Potential Benefits

- Dapagliflozin may reduce the risk of kidney failure after surgery, but this is not yet certain—that is why we are conducting this study.

Potential Risks or Drawbacks

- You may experience side effects or discomfort from the study medication.
- You may experience mild discomfort from the measurements conducted during the study.
- Participation will require extra time.
- You must adhere to study-related agreements and procedures.

What If You Choose Not to Participate?

Participation is entirely your decision. If you choose not to participate:

- You will receive standard treatment in case of acute kidney injury.
- This usually involves monitoring and waiting to see if kidney function recovers.
- If the kidney injury is severe, kidney replacement therapy (dialysis) may be required.

Your doctor can provide more details about the available treatment options, including their advantages and disadvantages.

**When Does the Study End?**

The researcher will inform you if new information becomes available that is important for you. At that point, you can decide whether you wish to continue participating.

Your participation in the study will end in the following cases:

- You have completed all study-related procedures as scheduled.
- You become pregnant during the study.
- You decide to withdraw from the study at any time. If you wish to stop, please inform the researcher immediately. You do not need to provide a reason. After withdrawing, you will receive the standard treatment and surgery. If you wish, the researcher may invite you for a final follow-up appointment.
- The researcher determines that it is better for you to stop. In this case, you may also be invited for a follow-up.
- The study is terminated by one of the following:
  - Amsterdam UMC (AMC)
  - The government
  - The medical ethics committee reviewing the study

What Happens If You Stop Participating?

- Any data and biological samples (such as blood) collected before you stopped will still be used in the study.
- If you wish to have your collected samples destroyed, please inform the researcher.
- The study will officially end once all 784 participants have completed the research.

**What Happens After the Study?**

*Can You Continue Using the Medication?*

*The study medication is designed to protect your kidneys during surgery, but there is no reason to continue taking it after surgery. However, your general practitioner or specialist may prescribe this medication in the future for other medical reasons.*

*Will You Receive the Study Results?*

*Approximately 12 months after the study is completed, the researcher can inform you about the key findings of the research. You may also be told which study group you were in.*

*If you wish to receive these results, please provide your email address at the end of the consent form.*

**What Happens to Your Data and Biological Samples?**

If you participate in the study, you consent to the collection, use, and storage of your data and biological samples.

What Data Do We Store?

We store the following information:

- Your name
- Your gender
- Your date of birth
- Your ethnicity
- Your health-related information
- Medical data collected during the study

What Biological Samples Do We Store?

We collect, use, and store blood samples for the study.

Why Do We Collect, Use, and Store Your Data and Samples?

- To answer the research questions of this study.
- To publish the study results.
- The study sponsor may analyze the research data.

How Do We Protect Your Privacy?

- Your data and biological samples are coded to protect your privacy.
- This code replaces your personal information, and only a secure key stored at the hospital can link the code back to you.
- Any study reports or publications will ensure that you cannot be identified.

Who Can Access Your Data?

Certain individuals may access your uncoded personal data, including:

- Study safety monitoring committee members
- Auditors working for the study sponsor
- National and international regulatory authorities

These individuals must keep your data confidential.

The Dutch Health and Youth Care Inspectorate (IGJ) can access your data without requiring your consent.

How Long Are Your Data and Samples Stored?

- Your data will be stored for 25 years at the hospital.
- Your blood samples will be stored for 2 years and will be destroyed once they are no longer needed.

Can Your Data and Samples Be Used for Other Research?

Your data and unused blood samples may also be useful for future research on kidney failure after heart surgery.

- If you agree, your data and samples will be stored for 2 years for potential future research.
- If you do not agree, you can still participate in this study and receive the same care.

Can You Withdraw Your Consent for Data Use?

Yes. You can withdraw your consent for data and sample use at any time. However:

- Previously collected data may still be used for the study.
- If your samples have already been analyzed, the results cannot be deleted but will not be used for future research.

Want to Know More About Your Privacy?

- More information on your rights: [www.autoriteitpersoonsgegevens.nl](http://www.autoriteitpersoonsgegevens.nl)
- If you have questions or complaints about your data, contact Amsterdam UMC (AMC) (see Appendix A).
- You may also file a complaint with the Data Protection Officer at AMC or the Dutch Data Protection Authority (Autoriteit Persoonsgegevens).

Where Can You Find More Information About the Study?

- Study details are available at [www.ClinicalTrials.gov](http://www.ClinicalTrials.gov).
- After the study ends, a summary of the results may be posted under study ID: NCT05590143.

**Will You Receive Compensation for Participation?**

No, participation in this study is voluntary and unpaid.
However, the study medication and additional tests are provided at no cost to you.

**Are You Insured During the Study?**

Yes. A special insurance policy has been arranged for all study participants.

- This insurance covers damages resulting from the study.
- However, not all damages are covered.
- See Appendix B for details on insurance coverage and how to report damages.

**Will Your Doctor and Pharmacist Be Notified?**

Yes.
The researcher will inform your doctor and pharmacist that you are participating in this study.
This is done for your safety.

**Do You Have Questions?**

If you have questions about the study, contact the researcher.

Would you like advice from someone independent who is not involved in the study?

- Contact the independent expert listed in Appendix A.

Do you have a complaint?

- Discuss it with the researcher or your doctor.
- If you prefer, you can contact the hospital’s complaints officer (see Appendix A).

**How Do You Give Your Consent?**

You will have time to think about whether you want to participate.

If you decide to participate:

- Inform the researcher that you understand the information.
- Sign the consent form provided with this information sheet.
- Both you and the researcher will keep a signed copy of the form.

Thank You for Your Time.

**Appendix A: Contact Information for the Study**

**X**

**Appendix B: Information About the Insurance**

**X**

**Appendix C: Study Procedure Schedule**

Before Hospital Admission:

- You will receive oral and written information.
- You will provide written consent to participate in this study.
- We will collect some medical data.

During Hospital Admission for Your Surgery:

- On the day before your surgery, you will receive the study medication.
- You will take the medication for the first time on the day before your surgery and then daily in the morning for the next three days. This means:
  - Once on the morning of your surgery
  - Once on each of the two days after surgery
- Around the time of your surgery, researchers will collect medical data. The only thing you will notice is that a blood sample will be taken once a day before the surgery and for seven days after the surgery to monitor your kidney function.

After Hospital Admission for Your Surgery:

- Thirty days after your surgery, we will send you a questionnaire.
- This questionnaire will ask about your experience during hospitalization and the weeks following your surgery.
- We will inquire about any side effects, quality of recovery, and possible complications.

**Appendix D: Informed Consent Form for Participants**

Behorende bij

Related to:
Dapagliflozin for the Reduction of Acute Kidney Injury After Heart Surgery – The MERCURI-2 Study
*proMoting Effective Renoprotection in Cardiac sURgery patients by SGLT2-Inhibitors*

- I have read the information letter. I had the opportunity to ask questions, and my questions have been answered sufficiently. I had enough time to decide whether I want to participate.
- I understand that participation is voluntary. I also understand that I can decide at any time to withdraw from the study without providing a reason.
- I give the researcher permission to inform my treating medical specialist and pharmacist that I am participating in this study.
- I give the researcher permission to request medical information from my general practitioner and the hospital where I was admitted before or after my heart surgery.
- I give the researcher permission to provide my general practitioner or treating specialist with any unexpected findings from the study that are relevant to my health.
- I give the researchers permission to collect and use my data and biological material solely to answer the research question of this study.
- I understand that, for the purpose of study oversight, certain individuals may access my full medical records. These individuals are listed in the information letter. I consent to them reviewing my data for this purpose.
- I understand that I must not become pregnant during the study.
- If applicable, the researcher has discussed with me the best ways to prevent pregnancy during the study.

**Would you please mark "Yes" or "No" in the table below?**

| I give permission to store my data for use in other research, as described in the information letter. | Yes ☐ | No ☐ |
| --- | --- | --- |
| I give permission to store my remaining biological material (blood) for use in other research, as described in the information letter. The biological material will be stored for 2 more years. | Yes ☐ | No ☐ |
| I give permission to be asked after this study if I would like to participate in follow-up research. | Yes ☐ | No ☐ |
| I give permission for the oxygen levels in my urine to be measured. | Yes ☐ | No ☐ |
| I give the researchers permission to inform me after the study regarding the treatment I received and which group I was in. | Yes ☐ | No ☐ |

**I want to participate in this research.**

My name is (participant): ……………………………………………………………..

Signature: ……………………………………………… Date : __ / __ / __

I declare that I have fully informed the participant about the research mentioned. If any information arises during the study that could affect the participant's consent, I will inform them in time.

Name researcher (or representative): …………………………………………………

Signature: ……………………………………………… Date: __ / __ / __

If you would like to know which group you were in and receive the results of the study after its completion, please fill in your email address here: ………………………………………………………@………………………………
