# Supplementary material for: Study protocol of the multicentre, randomised, triple-blind, placebo-controlled MERCURI-2 trial: promoting effective renoprotection in cardiac surgery patients by inhibition of sodium glucose cotransporter (SGLT)-2
Source: BMJ Open. 2025 May 16;15(5):e095504. doi: 10.1136/bmjopen-2024-095504 (PMC12086897; doi:10.1136/bmjopen-2024-095504)
Supplement: online supplemental file 2 [file bmjopen-15-5-s002.docx]

**Supplementary Table 1**. Recorded complications of the MERCURI-2 trial

| Complications |
| --- |
| Arrythmia |
| Myocardial infarction |
| Heart failure/ pleural effusion |
| Pericarditis/ pericardial effusion |
| Pneumothorax |
| Cerebrovascular stroke or haemorrhage |
| Sternal wound infections |
| Pneumonia |
| Sepsis/ bacteraemia |
| Urinary tract infections |
| Delirium |
| Coagulation disorders |
| Deep venous thrombosis/ pulmonary embolus |
| Re-operation |
| Complications already defined as secondary outcomes |
