# Supplementary material for: Study protocol of the multicentre, randomised, triple-blind, placebo-controlled MERCURI-2 trial: promoting effective renoprotection in cardiac surgery patients by inhibition of sodium glucose cotransporter (SGLT)-2
Source: BMJ Open. 2025 May 16;15(5):e095504. doi: 10.1136/bmjopen-2024-095504 (PMC12086897; doi:10.1136/bmjopen-2024-095504)
Supplement: Supplementary file 3 [file bmjopen-2024-095504.reviewer_comments.pdf]

PEER REVIEW HISTORY

BMJ Open publishes all reviews undertaken for accepted manuscripts. Reviewers are asked to complete a checklist review form and are provided with free text boxes to elaborate on their assessment. These free text comments are reproduced below.

ARTICLE DETAILS

Title (Provisional)

Study protocol of the multicenter, randomized, triple-blind, placebo-controlled MERCURI-2 trial: proMoting Effective Renoprotection in Cardiac sURgery patients by Inhibition of sodium glucose cotransporter (SGLT)-2

Authors

Oosterom-Eijmael, Maartina; Monteiro de Oliveira, Nelson P; Niesten, Ed D; Tolsma, Martijn; Snellen, Ferdinand TF; Voogd, Magiel F; Wink, Jeroen; van der Werff, Lisa MM; van Raalte, Daniel H; Gerritse, Bas M.; Scohy, Thierry V.; Rettig, T; Godfried, M B; Eberl, Susanne; Preckel, Benedikt; Hermanides, Jeroen; Hulst, Abraham H; Study group, MERCURI-2

VERSION 1 - REVIEW

**Reviewer**	**1**
**Name**	**Murphy, Gavin**
**Affiliation**	**University of Leicester College of Medicine Biological Sciences and Psychology, Cardiovascular Sciences**
**Date**	**12-Nov-2024**
**COI**	**None**

The authors do not include any description of the limitations of the trial;

1. AKI on its own is not considered an important clinical outcome; MAKE or some other outcome that includes a longer term assessment of the effects on kidney function are preferred.

2. Adverse event reporting is not described.

3. Important cardiovascular endpoints that are considered minimum reporting standards for cardiac surgery trials are not described.

4. The baseline stratification does not include baseline renal function, which would be expected.

5. The analysis plan will include Fischer's exact test; how will this incorporate baseline stratification variables

**Reviewer**	**2**
**Name**	**Irwin, MG**
**Affiliation**	**University of Hong Kong**
**Date**	**25-Feb-2025**
**COI**	**None**

This is a well designed and potentially very informative trial design that has important implications for clinical practice. You mention other medication use will be recorded - will there be any restrictions on this given certain drugs can have effects on perioperative renal function e.g. ARG, ACEI? What is the rational for starting dapagliflozin on the day of surgery rather than perhaps 5 days or a week before (I would have thought this would be better)? How will you manage patients who can't take oral medication after cardiac surgery (this is not unusual)? Can you clarify whether anaesthesia and analgesia will be standardised? Will NSAID be avoided, for example. The same applies to fluid therapy e.g. crystalloid (type and amount) v colloid?

VERSION 1 - AUTHOR RESPONSE

Reviewer: 1
Dr. Gavin Murphy, University of Leicester College of Medicine Biological Sciences and Psychology
Comments to the Author:
The authors do not include any description of the limitations of the trial;
1. AKI on its own is not considered an important clinical outcome; MAKE or some other outcome that includes a longer term assessment of the effects on kidney function are preferred.

Thank you for reviewing our manuscript. We have included in the strengths and limitations section that a limitation of the study is that the primary outcome is postoperative AKI and not a longer term outcome. We chose AKI as primary outcome as it was the most feasible outcome measure for the conduct of this multicenter trial. And collect AKI outcomes as advised in the consensus statement of the 31st Acute Disease Quality Initiative (ADQI). (Reference: Recommendations for clinical trial design in acute kidney injury from the 31st acute disease quality initiative consensus conference. A consensus statement - PMC) We will also report outcomes included in the MAKE30 such as kidney related death, number of patients requiring new dialysis and patient with a worsened kidney failure at day 30, to clarify this, we elaborated the text on P9.
Another validated clinically important secondary endpoint we included is DAH30. (Reference: Days at Home after Surgery: An Integrated and Efficient Outcome Measure for Clinical Trials and Quality Assurance - eClinicalMedicine and Validation of days at home as an outcome measure after surgery: a prospective cohort study in Australia - PMC) With this, we aimed to design a feasible multicenter trial with relevant clinical outcomes.

Page 9:
“The primary outcome of this study is the incidence of AKI according to the KDIGO criteria (Table 2) based on serum creatinine and urine output. Serum creatinine levels and urine output will be reviewed by a second investigator and the study monitor to ensure primary outcome adjudication.

Other secondary kidney-related outcome parameters include the difference in the primary outcome between males and females, incidence of individual AKI stages according to KDIGO criteria, the maximum change of creatinine postoperative compared to the baseline creatinine preoperative, the postoperative creatinine course, the number of patients with a persistent kidney failure (defined as creatinine concentration ≥ 200% of baseline creatinine concentration after 30 days), the number of patients requiring dialysis, the number of patients who died due to a primary kidney-related cause."

2. Adverse event reporting is not described.

As you suggested, we added a paragraph about adverse event reporting.

Page 10:

"Regarding serious adverse events (SAE's), the sponsor will yearly present an overview of the SAE's via CTIS. Suspected unexpected serious adverse reactions (SUSARs) will be reported earlier via EudraVigilance depending on the seriousness of the reaction and will be as follows: In the case of fatal or life-threatening SUSARs, as soon as possible and in any event not later than seven days after the sponsor became aware of the reaction, in the case of non-fatal or non-life-threatening SUSARs, not later than fifteen days after the sponsor became aware of the reaction and In the case of a SUSARs which was initially considered to be non-fatal or nonlife threatening but which turns out to be fatal or life-threatening, as soon as possible and in any event not later than seven days after the sponsor became aware of the reaction being fatal or life-threatening."

3. Important cardiovascular endpoints that are considered minimum reporting standards for cardiac surgery trials are not described.

We acknowledge that the cardiovascular endpoints were not clearly presented in our manuscript, and have now revised the text to include them more clearly. Supplementary table 1 lists the recorded complications after cardiac surgery.

Page 9:
"The cardiac-related secondary outcomes include the incidence of de novo postoperative atrial fibrillation, registered on a 12-lead ECG, the maximum postoperative creatine kinase (CK)-MB or troponine concentrations, the number of patients who developed new onset cardiac arrhythmia, new myocardial infarctions or cerebrovascular accident confirmed by computed tomography (CT)-scan wihtin the first 30 days postoperative, as well as the number of patients who required an extended hospital stay due to heart failure, needed readmission for heart failure, or died from a primary cardiac cause. Furthermore, length of stay in the Intensive Care Unit and the hospital measured in days and all cause mortality were collected (Table 3)."

4. The baseline stratification does not include baseline renal function, which would be expected.

In this study, we included stratification before randomization based on two dichotomous baseline characteristics: sex and type 2 diabetes. We chose not to stratify by baseline kidney function, as it is not a dichotomous variable. Instead, we have planned a post hoc analyses to assess the potential

influence of baseline kidney function on the treatment effect of SGLT2 inhibitors. This approach will allow us to explore the relationship between the treatment effect and kidney function without loss of information that could occur by converting a non-dichotomous variable into a dichotomous one.

5. The analysis plan will include Fischer's exact test; how will this incorporate baseline stratification variables

Stratification for sex and type 2 diabetes before randomization will be incorporated. This stratification ensures that treatment and placebo groups are balanced in these two key variables. Our analysis plan includes a Fisher's exact test for the primary outcome: AKI. Additionally, post hoc analyses with regression analyses and interaction terms will assess the influence of other baseline characteristics - including age, BMI, baseline kidney function and heart failure - on the treatment effect of SGLT2 inhibitors. We agree that it might not be clear that Fishers exact test will be used for the primary outcome but post hoc analyses will be conducted with regression analyses, to improve clarity we changed the manuscript.

Section secondary study outcome P12:

"Predefined post hoc analysis with regression analyses will be conducted to estimate the influence of age, BMI, baseline kidney function and the presence of heart failure on the treatment effect and their possible interactions on the primary outcome."

Reviewer: 2
Dr. MG Irwin, University of Hong Kong
Comments to the Author:
This is a well designed and potentially very informative trial design that has important implications for clinical practice. You mention other medication use will be recorded - will there be any restrictions on this given certain drugs can have effects on perioperative renal function e.g. ARG, ACEI? What is the rational for starting dapagliflozin on the day of surgery rather than perhaps 5 days or a week before (I would have thought this would be better)? How will you manage patients who can't take oral medication after cardiac surgery (this is not unusual)? Can you clarify whether anaesthesia and analgesia will be standardised? Will NSAID be avoided, for example. The same applies to fluid therapy e.g. crystalloid (type and amount) v colloid?

Thank you for reviewing our manuscript. We also believe that our results may have important implications for clinical practice. We do record the use of medication such as ARB and ACEI; however, their use is not restricted. Additionally, in this trial, we do not standardize anesthesia, analgesia or fluid therapy. According to the guidelines, NSAIDs will be avoided in the cardiac surgery population and other pain killers will be used. We will avoid influencing normal clinical practice, allowing us to evaluate the effect of treatment in a real-world setting. The guidelines for clinical practice will be followed and our intervention will be on top of standard of care. Our rational for initiating dapagliflozin one day before surgery, rather than 5 or 7 days in advance, is primarily logistical. In the Netherlands, surgical planning is finalized only one day before the procedure. If our treatment strategy proves effective, it will also be very easily implementable in clinical practice. Study participants who are unable to take oral medication after cardiac surgery will not be able to receive the study medication. If this strategy were to be implemented in clinical practice, patients could receive SGLT2 inhibitors via a feeding tube. However, this is not feasible within the study setting, as crushing the tablets would compromise blinding.

VERSION 2 - REVIEW

Reviewer 1

Name Murphy, Gavin

Affiliation University of Leicester College of Medicine Biological Sciences and Psychology, Cardiovascular Sciences

Date 23-Apr-2025

COI

I am not convinced that a chi squared test is appropriate for the primary outcome evaluation but this has been justified by the investigators.

Reviewer 2

Name Irwin, MG

Affiliation University of Hong Kong

Date 16-Apr-2025

COI

Thank you for answering my questions satisfactorily and I wish you success with your trial
